# Treatment of paediatric pontine glioma with oral trophosphamide and etoposide

**DOI:** 10.1038/sj.bjc.6600552

**Published:** 2002-10-21

**Authors:** J E A Wolff, S Westphal, G Mölenkamp, A Gnekow, M Warmuth-Metz, D Rating, J Kuehl

**Affiliations:** St. Hedwigs Klinik, Hämato/Onkologie, Steinmetzstr. 1–3, 93049 Regensburg, Germany; Department of Oncology, Klinik für Kinder und Jugendliche, K.Z.V.A. Stenglinstr. 2, 86156 Augsburg, Germany; Brain Tumor Research Centre, Montreal Neurological Institute, 3801 University Street, Montreal, QC, Canada H3A 2B4; Department of Radiology, Universitaets-Kinderklinik, Josef-Schneider-Str. 2, 97080 Wuerzburg, Germany; Department of Pediatric Neurology, University Hospital, Im Neuenheimer Feld, Heidelberg, Germany; Pediatric Oncology, Universitaets-Kinderklinik, Josef-Schneider-Str.2, 97080 Wuerzburg, Germany

**Keywords:** VP16, trophosphamide, children, brain tumours, brain stem glioma

## Abstract

To evaluate the overall survival of paediatric patients with pontine gliomas treated with oral trophosphamide and etoposide. Patients between 3 and 17 years of age with either typical diffuse pontine glioma on MRI or histologically proven anaplastic astrocytoma/glioblastoma multiforme located in the pons, were eligible. Treatment consisted of oral trophosphamide 100 mg m^−2^ day^−1^ combined with oral etoposide at 25 mg m^−2^ day^−1^ starting simultaneously with conventional radiation. Twenty patients were enrolled (median age 6 years, male : female=9 : 11). Surgical procedures included: no surgery: five, open biopsy: three, stereotactic biopsy: six, partial resection: three, and sub-total resection: three. Histological diagnoses included pilocytic astrocytoma: one, astrocytoma with no other specification: three, anaplastic astrocytoma: three, glioblastoma multiforme: eight, no histology: five. The most frequent side effects were haematologic and gastrointestinal. There was no toxic death. The response to combined treatment in 12 evaluable patients was: complete response: 0, partial response: three, stable disease: four, and progressive disease: five. All tumours progressed locally and all patients died. The overall median survival was 8 months. The overall survival rates at 1 and 4 years were: 0.4 and 0.05 respectively. This was not different from a control group of patients documented in the same population. Oral trophosphamide in combination with etoposide did not improve survival of pontine glioma patients. The treatment was well tolerated and should be evaluated for more chemoresponsive paediatric malignancies.

*British Journal of Cancer* (2002) **87**, 945–949. doi:10.1038/sj.bjc.6600552 www.bjcancer.com

© 2002 Cancer Research UK

## 

The term ‘brain stem glioma’ had originally developed as a clinical diagnosis without histological confirmation because the morbidity for surgical intervention within the pons was high and the relevance of histological diagnoses was low. By now, MRI morphology differentiates at least three groups of tumours to be treated in very different ways (Barkovich *et al*, 1990, 1991; Albright *et al*, 1993; Fischbein *et al*, 1996). (i) The dorsally exophilic cervicomedullary tumours appear to benefit significantly from surgical resection (Epstein and Constantini, 1996). (ii) Intrinsic tectumglioma may be associated with a long history and with neurofibromatosis type I. In this case they grow very slowly (Pollack *et al*, 1996; Bilaniuk *et al*, 1997), and a ‘wait-and-see’ approach can provide the patient with long duration of good quality of life. (iii) Intrinsic diffused pontine glioma, in contrast, have a poor prognosis (Freeman and Farmer, 1998). The radiological characteristics of these tumours are: Pontine location, hypodensity on CT, hypointensity on T1 MRI images, hyperintensity on T2 images, no contrast enhancement, and almost complete involvement of the cross-section of the pons. The tumours occur in the first decade of life after a short history and progress quickly after diagnosis. These patients are treated with radiotherapy, which is frequently combined with experimental approaches. While the MRI images appear to be highly predictive in these three groups of tumours, there are other, less typical lesions in which imaging technologies have not been suitable to predict the prognosis. Among those are focal, cystic, and contrast enhancing pontine lesions. Many of those will, on histology, turn out to be high grade glioma, but other tumour histologies, inflammations, and histiocytic lesions (Weaver *et al*, 1999) are possible too. To make matters more complicated, diffuse pontine glioma can turn into cystic or focally contrast enhancing lesions as a part of their natural history (Nelson *et al*, 1994).

On histology, brain stem gliomas are predominately astrocytic tumours. In other locations and age groups, tumour gradings such as the WHO classification, have proven to be of high prognostic value. This even includes adult patients with brain stem glioma (Selvapandian *et al*, 1999). But there appears to be a biologically different tumour entity in the paediatric age group of pontine glioma. In this age group, histological grading has not been of prognostic significance (Steck and Friedman, 1995; Selvapandian *et al*, 1999; Kaplan *et al*, 1996). Two distinct age peaks in the incidence of brain stem glioma (first decade and third to fourth decade of life) support this hypothesis of biologically different tumours (Tokuriki *et al*, 1986; Steck and Friedman, 1995; Selvapandian *et al*, 1999). Diffuse pontine glioma in children remains almost unique as a diagnosis, in which radiation and intensive chemotherapy is done without molecular or histological diagnosis (Albright *et al*, 1993; Allen *et al*, 1999).

Radiotherapy frequently results in clinical improvement of diffuse pontine glioma and approximately doubles the median survival to 1 year (Freeman and Farmer 1998). Increasing the intensity of radiotherapy by hyperfractionation (Packer *et al*, 1994, 1996), and acceleration (Lewis *et al*, 1997) did not increase the survival further and the sequelae suggest caution with this approach (Freeman *et al*, 1996). Chemotherapy did not add to the therapeutical success of radiation treatment. In contrast to the success in medulloblastoma, the addition of CCNU, vincristine, and prednisone did not improve survival in a prospective randomised trial (Jenkin *et al*, 1987). Intensifying nitrosurea treatment with stem cell rescue (Bouffet *et al*, 1997) or the use of other bone marrow transplant agents such as thiotepa, VP16, busulphan, and carboplatin (Dunkel *et al*, 1998; Kalifa *et al*, 1999) did not improve survival either. Presently, novel agents such as topotecan (Kadota *et al*, 1999), temozolomide (Estlin *et al*, 1998) and simultaneous chemotherapy with radiation (Allen *et al*, 1999) are studied.

In 1988 the Society of Pediatric Oncology and Hematology in the Germanic language group (GPOH) started to document (HIT-DOC) paediatric brain tumour patients who are not enrolled in other clinical studies. For patients with diffuse pontine glioma and glioblastoma multiforme, a series of phase I/II treatment trials was activated in 1995 (Wolff *et al*, 1996a). This report describes the final data of the first of these trials (HIT-GBM-A) for pontine glioma. In this study, patients were treated with conventional radiotherapy and oral chemotherapy with trophosphamide 100 mg m^−2^, and etoposide 25 mg m^−2^ daily^−1^ (Wolff *et al*, 1996b). The results of malignant glioma in location outside of the brain stem has been reported elsewhere (Wolff *et al*, 2000).

## PATIENTS OF THE TREATMENT GROUP

This treatment protocol was open for patients between 3 and 17 years of age, with newly diagnosed pontine tumours. The diagnosis had to be confirmed by at least one of two reference centres. Typical diffuse pontine gliomas were enrolled based on the characteristic MRI morphology in combination with a history of less than 6 months. The MRI morphology had to be confirmed by the Neuroradiology Reference Center. Patients with atypical radiomorphology required a histological confirmation. In those patients, only anaplastic astrocytoma grade III (WHO) and glioblastoma multiforme were enrolled. The histology had to be confirmed by the Neuropathology Reference Center. The study enrolled patients mainly between July 1995 and April 1997, by which time it was substituted by the following protocol (‘HIT-GBM-B’). However, two patients were enrolled after the planned study closure and were not excluded from this analysis.

Twenty patients with pontine tumours were enrolled in the treatment study HIT-GBM-A. The male : female ratio was 9 : 11, the median age was 6 years (Table 1

**Table 1:**
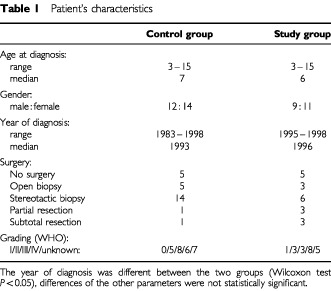
Patient's characteristics

). The family history and previous history of the patients were unremarkable in all. All tumours were located in the pons, there was no dorsal exophytic tumour in this group. Surgery was done in 15 of the 20 patients (Table 1). Only nine of these patients would have required histology for enrollment because the tumours were contrast enhancing, the other six patients who had undergone surgery had typical radiomorphology. Surgical procedures included stereotactic biopsy in six, open biopsy in three, partial resection in three, and sub-total resection in three patients. Histology included: three astrocytomas with no other specifications WHO grade II, one pilocytic astrocytoma, three anaplastic astrocytomas, and eight glioblastoma multiformes (Table 1). The tumours with grade I and grade II histology were enrolled based on the MRI morphology of diffuse pontine glioma.

## PATIENTS OF THE CONTROL GROUP

The documentation study ‘HIT-DOC’ allowed retrospective enrollment. In contrast to the treatment study, a Neuroradiology Reference Centre did not exist for the patients of the documentation study. In order to compile a control group compatible with the treatment group, the following inclusion criteria were used: location=‘pons’, age=3–17 years, and radiation=‘yes’. Exclusion criteria were: (i) double-documentation on the treatment cohort, and (ii) patients enrolled in one of the later treatment protocols (HITGBM-B, and -C). This left 26 patients eligible for the control group. As expected, the year of diagnosis was much more widespread, ranging from 1983 to 1998 (Table 1). The age at diagnosis, however, was quite similar to the treatment group (median age: 7 years, range 3–17, Table 1). The male : female ratio was 12 : 14. Surgery was performed in 21 of these patients and was classified as: stereotactic biopsy: 14, open biopsy: five, partial resection: one, sub-total resection: one (Table 1). Histological results were diagnostic in 19 patients: Two fibrillary astrocytomas, three astrocytomas with no other specification WHO grade II, eight anaplastic astrocytomas, and six glioblastoma multiformes (Table 1).

## POSTOPERATIVE TREATMENTS

The study protocol included up front standard fractionated radiotherapy to the tumour region plus 2 cm margins in 30 fractions of 1.8 Gy resulting in a total dose of 54 Gy, given over a period of 6 weeks with one radiation on 5 of 7 days per week. This was completed in 18 of the 20 patients. A 5-year-old girl received only 50 Gy after a partial resection of a glioblastoma multiforme, an 8-year-old boy with stereotactically biopsied pilocytic astrocytoma received 56 Gy.

The chemotherapy included trophosphamide 100 mg m^−2^ day^−1^ per os, and etoposide 25 mg m^−2^ day^−1^ per os daily for 21 days followed by a week of rest, after which the next cycle started. Chemotherapy was started simultaneously with radiation as soon as the diagnosis was confirmed and was planned for a period of 1 year or 13 cycles. All patients of the treatment group started this protocol. It was discontinued when the tumours progressed, and it was extended after completing the 13 cycles in a few patients by parents'/physicians' choice. This resulted in a mean number of cycles of 7.2 (standard deviation 5.4, median 6.5, range 1–19).

The control group's adjuvant treatment was rather inhomogeneous, as it was not a treatment protocol but a documentation study, summarising, what was done by different institutions. By definition, all patients had radiotherapy. Five patients had stereotactic seed implantations, while three of them had additional external radiotherapy. The external radiotherapy doses varied between 15 and 64.8 Gy (mean 46.6, standard deviation 14.5, median 54 Gy). In addition to the radiation, chemotherapy was given in 16 patients. Three patients received carboplatinum/vincristine, one patient was treated according to the CCNU/vincristine/cisplatinum chemotherapy protocol (Packer *et al*, 1996, 1999), four patients were treated according to an intense chemotherapeutic protocol designed for medulloblastoma, using cisplatinum, Ara-C, etoposide, iphosphamide, and high dose methotrexate (Kuhl *et al*, 1998), and six patients received various types of other institutional chemotherapeutic protocols. Ten patients did not receive any chemotherapy, and chemotherapy was not documented in one patient.

## RESULTS

**Figure 1:**
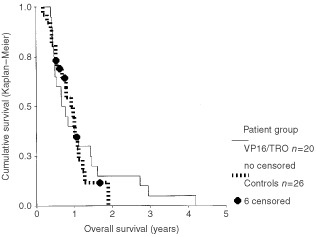
Overall survival of pontine glioma patients treated with etoposide and trofosfamide (VP16/TRO, *n*=20), compared to patients with other treatments (controls, *n*=26). Six patients of the control group were lost to follow up (six censored). The difference between the two groups is not statistically significant (log rank test *P*=0.7).

Oral chemotherapy with trophosphamide/VP16 was well tolerated. There were no treatment-related deaths. Less severe side effects were not the primary objective of this study, and the documentation was insufficient in four patients. Of the remaining 16 patients, one had no side effects. Nausea was reported in eight patients, and vomiting in five of those. Haematological side effects were reported in 13 patients (NIH grade II: eight, grade III: two, grade IV: three). Of those, one patient developed fever and neutropenia and required admission for antibiotic treatment. Rare side effects included increase of liver enzymes (NIH grade III), abdominal pain, dysuria (NIH grade II), and haematuria (NIH grade I) in one patient each. Response to treatment could be evaluated in 12 patients. The response rates were: complete response: 0, partial response: three, stable disease: four, progressive disease: five. All patients eventually died from local tumour progression. Time to progression was defined as time interval between diagnosis and either tumour growth seen in neuroimaging (>25% product of two perpendicular diameters) or death of disease, whatever came first. Using this definition, the median time to progression was 5.9 months (standard error: 1.7 months, 95% Confidence Interval (CI): 2.6–9.1), the mean time to progression was 9.6 months (standard error: 1.9, 95%CI: 5.8–13.4). The median overall survival time was 8 months (standard error 1.6, 95%CI: 4.9–11), the mean overall survival time was 13.8 months (±2.8, 95%CI: 8.4–19.2). The overall survival rates (and standard errors) at 1, 2, 3, 4, and 5 years were: 0.4 (±.11), 0.15 (±.08), 0.05 (±.05), 0.05 (±.05), and 0 (±0), (Figure 1

).

The control group from the documentation study (HIT-DOC) had comparable survival rates with a median event free survival time of 7 months (standard error 2.8, 95%CI 1.7–12.2). The median overall survival time of the control group was 11 months (standard error 1.6, 95%CI: 7.8–14.2). The 1 and 2 year overall survival rates were 0.40 (±0.11 standard error), and 0 (±0). The survival curves (Figure 1) of the two groups were superimposeable up to 1 year of follow up. After that, the VP16/trophosphamide group seems to have a slightly longer overall survival. However, this was not significant (*P*=0.7 for overall survival, *P*=0.75 for progression-free survival, definitions identical in both patient groups, log rank tests). Multivariate analyses done with the Cox regression model, with both overall and progression free survival as endpoints, confirmed the lack of a significant difference between the two groups.

## DISCUSSION

Trophosphamide is an oxazaphosphorine, which is more lipid soluble as compared to cyclophosphamide and iphosphamide. This results in better intestinal reabsorption and allows oral treatment. Hepatic activation produces 4-hydroxy metabolites, including 4-hydroxy-iphosphamid (Boos *et al*, 1993). While other oxazaphosphorines are widely used in the treatment of astrocytic tumours (Freeman and Perilongo, 1999), trophosphamide (Wagner *et al*, 1997) had not yet been investigated in brain tumours to our knowledge. It has shown efficacy in soft tissue sarcoma (Blomqvist *et al*, 1995; Kollmannsberger *et al*, 1999), malignant lymphoma (Helsing, 1997), and non-Hodgkin lymphoma (Salminen *et al*, 1997). Negative results were published in colorectal cancer (Strumberg *et al*, 1997) and pancreatic cancer (Schmidt-Sandte *et al*, 1999).

Etoposide (VP16) is a topoisomerase II inhibitor from the group of epipodophyllotoxins. It is widely used in paediatric brain tumour treatment and given in many different schedules. Oral continuous chemotherapy has recently shown activity in glioma (Chamberlain, 1997; Needle *et al*, 1997). Combining DNA alkylating agents with topoisomerase inhibitors is frequent practice and partially based on the rationale, that the topoisomerases are necessary for DNA repair mechanisms activated after DNA alkylation. Unfortunately, this effect also seems to enhance haematological side effects, which increase upon combining the two agents. Therefore, in combinations, drug dosages need to be reduced.

Treatment intensification has increased cure rates in a number of paediatric malignancies. In phase II studies, normally one dose level below the maximal tolerated dose is used. In view of the short remaining lifetime of patients with pontine glioma, the trophosphamide/VP16 treatment intensity was picked differently (Tillmann *et al*, 1998). The long term schedule was chosen similar to leukaemia maintenance chemotherapy and antiangiogenic chemotherapeutic approaches. The plan with respect to treatment intensity was to avoid the need for inpatient treatment, and this goal was achieved in all but one patient, who was admitted for fever and neutropenia. Less severe side effects such as nausea/vomiting, and leukopenia occurred in all but one patient, indicating that the dose was not too low. Judged by this measure, the chemotherapeutic dosing was adequate, and can be recommended for phase II studies, when the same intensity is desired in the treatment of other paediatric malignancies. Higher doses are obviously possible, but will likely increase the number of inpatient admissions for supportive care.

Diffuse and malignant pontine glioma in childhood remains a challenge of paediatric cancer care. Our treatment did not change this, as it was largely without effect on this tumour (Figure 1). The survival curve could suggest a benefit by increasing the remaining life span for about 20% of the patients. However, this is not statistically different from the control group. Since this was not a formal randomised phase III study, there is some risk that an existing minor benefit may not be detected. In addition, the control group is somewhat inhomogeneous since some patients received chemotherapy far more intense than the oral trophosphamide/VP16, which was tested here. However, the population available for registration of patients was not large enough for a phase III study. The prospective cohort comparison study design comparing each protocol with the previous cohorts, was the best that could be done in that situation. Trophosphamide/VP16 was the first of these protocols, the evaluation of which has to rely on a comparison with data collected in another setting. The German documentation study (HIT-DOC) was the most appropriate set of data for this, since they were collected in the same population and partially covered the same period for years of diagnosis. In this comparison, no significant difference was detected between the two groups.

Response to chemotherapy is a difficult endpoint for a chemotherapy study with paediatric pontine glioma. Up to now, no chemotherapeutic protocol has matched the results of radiation. Delaying radiation in order to collect clean data for response to chemotherapy, had been controversially discussed and would have reduced the number of participating centres. The concept might be more suitable for a disease, which does not progress as fast as pontine glioma. Starting chemotherapy simultaneously with radiation will give response rates to the combined treatment. These data are of limited value unless they are compared to response rates of radiation only. In this cohort, the response of 12 patients was: 0/3/4/5 for complete response/partial response/stable disease/progressive disease. When comparing this to literature data from other populations, there does not appear to be an improvement (Packer *et al*, 1994; Lewis *et al*, 1997). Since survival times were not prolonged either (Figure 1), these data suggest that the oral chemotherapy with TRO/VP16 did not add to the antitumour effect of radiotherapy.

The experience of this study with pontine glioma patients is negative with respect to antitumour effects. This does not preclude antitumour effectiveness in other malignancies, since all chemotherapeutic protocols tested in pontine gliomas up to now have failed, even when they are effective in more chemoresponsive tumours. This combination can safely be given to paediatric patients as palliative chemotherapy in other settings, since it was easy to monitor, side effects were mild, and since the treatment intensity can easily be adjusted on an individual basis.
